# A randomized open-label clinical trial on the effect of Amantadine on post Covid 19 fatigue

**DOI:** 10.1038/s41598-024-51904-z

**Published:** 2024-01-16

**Authors:** Ali Amini Harandi, Hossein Pakdaman, Aida Medghalchi, Negin Kimia, Alireza Kazemian, Fatemeh Siavoshi, Siavash Shirzadeh Barough, Akram Esfandani, Mohammad Hossein Hosseini, Seyed Ali Sobhanian

**Affiliations:** 1 Brain Mapping Research Center, Shahid Beheshti University of Medical Sciences, Tehran, Iran; 2 Pharmacy Department, Tehran Medical Sciences, Islamic Azad University, Tehran, Iran

**Keywords:** Medical research, Neurology, Infectious diseases, Viral infection

## Abstract

Many COVID-19 survivors experience lingering post-COVID-19 symptoms, notably chronic fatigue persisting for months after the acute phase. Despite its prevalence, limited research has explored effective treatments for post-COVID-19 fatigue. This randomized controlled clinical trial assessed the impact of Amantadine on patients with post-COVID-19 fatigue. The intervention group received Amantadine for two weeks, while the control group received no treatment. Fatigue levels were assessed using the Visual Analog Fatigue Scale (VAFS) and Fatigue Severity Scale (FSS) questionnaires before and after the trial. At the study's onset, VAFS mean scores were 7.90 ± 0.60 in the intervention group and 7.34 ± 0.58 in the control group (*P*-value = 0.087). After two weeks, intervention group scores dropped to 3.37 ± 0.44, significantly lower than the control group's 5.97 ± 0.29 (*P*-value < 0.001). Similarly, FSS mean scores at the trial's commencement were 53.10 ± 5.96 in the intervention group and 50.38 ± 4.88 in the control group (*P*-value = 0.053). At the trial's end, intervention group scores decreased to 28.40 ± 2.42, markedly lower than the control group's 42.59 ± 1.50 (*P*-value < 0.001). In this study, we report the safety, tolerability, and substantial fatigue-relieving effects of Amantadine in post-COVID-19 fatigue. The intervention demonstrates a statistically significant reduction in fatigue levels, suggesting Amantadine's potential as an effective treatment for this persistent condition.

## Introduction

The severe acute respiratory syndrome coronavirus 2 (SARS-CoV-2) has severely affected the health of the general public entire world. Vaccination decreased the overall incidence and severity of COVID-19. However, many patients complain of symptoms including fatigue, cough, shortness of breath, and muscle pain long after recovering from the acute phase of the disease^1^.

Persistent symptoms of COVID-19 are known with different terms, mainly post-COVID-19 syndrome^2,3^. These post-COVID-19 symptoms can be seen in patients regardless of their disease severity in the acute phase^4,5^, therefore, it can be an additional heavy burden on the health system^2^. Similar to the condition of post-viral syndrome that was seen after two years in survivors of SARS-COV-19^6^.

﻿Although chronic fatigue is the most common and persistent symptom which can last even months after recovery from COVID-19^7–10^ the mechanism of post-COVID-19 fatigue is still ambiguous. Several pathways have been hypothesized to contribute to post-COVID-19 fatigue. Dysfunction of the glymphatic system^11^ and muscle mitochondria, dysregulation of the neurotransmitters, and inflammation of the brain parenchyma^12^ seem to be associated with fatigue.

Based on the similarities between chronic fatigue syndrome (CFS) of viral infections^13,14^ and neurological symptoms of post-COVID-19 the treatment approaches for CFS could be considered for post-COVID-19 fatigue. However, non-pharmacological therapies, including cognitive behavior therapy, graded-exercise-related therapies, rehabilitation, acupuncture, and pharmacological treatments like antidepressants, have shown inconsistent results^15–19^ in CFS.

Because of the ambiguous pathophysiology of post-COVID-19 fatigue, suggesting a potential pharmacological treatment remained challenging. However, based on the promising effectiveness of Amantadine in reducing fatigue related to Multiple Sclerosis (MS)^20,21^ in previous studies, it is worthy to be evaluated as a potential treatment for post-COVID-19 fatigue.

This open-label study aims to investigate the effect of Amantadine on patients with post-COVID-19 fatigue using﻿ the Fatigue Severity Scale [FSS] which is one of the most commonly used scales for measuring fatigue in clinical trials^22^ as well as Visual Analog Fatigue Scale (VAFS).

## Results

### Demographic and baseline characteristics of the participants

**Figure 1:**
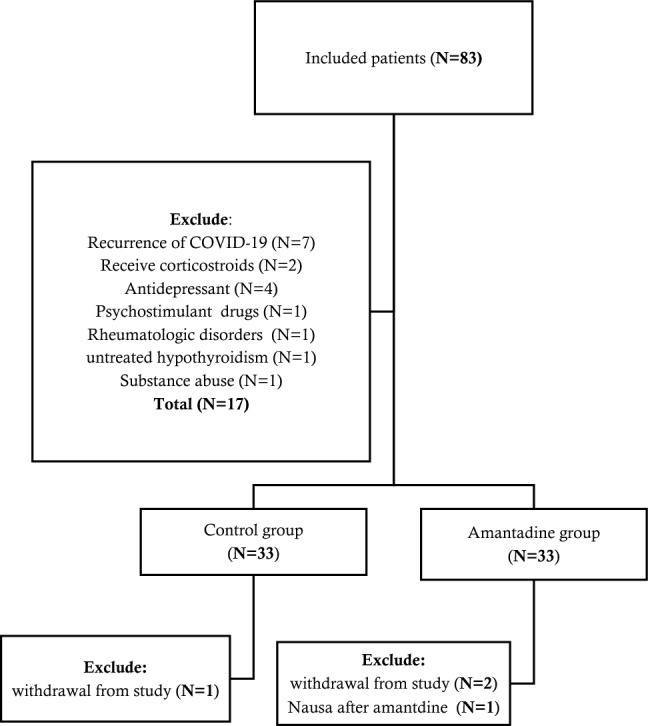
Participant flow diagram.

As shown in Fig. 1, of the total 66 recruited patients at the beginning of the study, one patient stopped taking Amantadine because of severe nausea and abdominal pain, and three patients (two patients from the Amantadine group and one patient from the control group) who lost to follow-up were excluded from the study, and 62 patients completed the study. The two groups were matched at baseline in terms of sex and history of hospitalization due to COVID-19. Among the 62 evaluated patients, 23 (37.10%) were male and 39 (62.90%) were female. The average age of the patients was 37.31 ± 8.98 years. Nearly half of the patients in each group had a history of hospitalization due to the most recent COVID-19 infection with no significant difference (*P*-value: 0.585). Demographic data and baseline characteristics of the participants are given in detail in Table 1. The mean of baseline VAFS and FSS scores in the Amantadine and control groups were not different from each other significantly (*P*-value = 0.087, and *P*-value = 0.053, respectively) (Table 2).

**Table 1:** Demographic characteristics of the patients. continuous variables were statistically compared using an independent *t*-test and categorical variables were compared between two groups using a chi-square test.

Basic information	Total (n = 62)	Amantadine (n = 30)	Control (n = 32)	*P*-value
Sex (Percentage (number))				
Female	62.90% (39)	63.33% (19)	62.50% (20)	0.945
Male	37.10% (23)	36.67% (11)	37.50% (12)	
Age (mean ± SD)	37.31 ± 8.98	36.16 ± 1.52	38.42 ± 1.68	< 0.0001
BMI (mean ± SD)	26.52 ± 4.43	25.78 ± 0.75	27.31 ± 0.82	< 0.0001
Hospitalization (Percentage (number))				
Yes	43.55% (27)	40.00% (12)	46.87% (15)	0.585
No	56.45% (35)	60.00% (18)	53.13% (17)	

*BMI* body mass index.

**Table 2:** Comparison of fatigue scores of the case and control groups measured using VAFS and FSS questionnaires using independent t-test.

Fatigue scoring scales	Before treatment (Mean ± SD)	After treatment (Mean ± SD)	Difference between before and after treatment (Mean ± SD)
VAFS			
Amantadine group	7.90 ± 0.60	3.37 ± 0.44	4.53 ± 0.44
Control group	7.34 ± 0.58	5.97 ± 0.29	1.37 ± 0.13
*P*-value	0.087	< 0.001	< 0.001
FSS			
Amantadine group	53.10 ± 5.96	28.40 ± 2.42	24.70 ± 7.20
Control group	50.38 ± 4.88	42.59 ± 1.50	7.79 ± 0.86
* P*-value	0.053	< 0.001	< 0.001

*VAFS* visual analogue fatigue scale, *FSS* fatigue severity scale.

### Comparison of VAFS in Amantadine and control groups

The results of the VAFS scores before and after treatment in the two groups are shown in Table 2. The mean and standard deviation of VAFS score at the beginning of the study in the Amantadine group was 7.90 ± 0.60 and in the control group was 7.34 ± 0.58; after two weeks of treatment, they decreased to 3.37 ± 0.44 and 5.97 ± 0.29, respectively (*P*-value < 0.001). Furthermore, patients in the Amantadine group had a significantly greater reduction in the VAFS scores compared to the control group (*P*-value < 0.001).

### Comparison of FSS in Amantadine and control groups

The changes in FSS scores after two weeks are shown in Table 2. In the Amantadine group, the FSS score decreased significantly. The mean and standard deviation of the baseline FSS score at the beginning in the Amantadine group was 53.10 ± 5.96 and in the control group was 50.38 ± 4.88; after two weeks, they decreased to 28.40 ± 2.42 and 42.59 ± 1.50, respectively (*P*-value < 0.001). Furthermore, patients who received Amantadine had a significantly greater reduction in the FSS scores compared to the control group (*P*-value < 0.001).

### Amantadine side effects

All reported side effects were transient and tolerable for patients, except for one who stopped taking the drug due to severe nausea and abdominal pain and subsequently was excluded from the study. According to Table 3, the most commonly reported side effect was dry mouth (26.67%).

**Table 3:** The prevalence of common side effects of drug use in the Amantadine group.

Side effects	Percentage (number)
Headache	13.33% (4)
Dizziness	16.67% (5)
Dry mouth	26.67% (8)
Seizure	0.00% (0)
Peripheral edema	0.03% (1)
Total	33.33% (10)

## Discussion

In this study, we investigated the effect of Amantadine on patients with post-COVID-19 chronic fatigue. Our results showed a significant difference in the improvement of fatigue in patients receiving Amantadine compared to the control group. This improvement was evident in the FSS and the VAFS scores since the average of both reached less than half of the initial value after two weeks of treatment with Amantadine.

Getting post-COVID-19 syndrome is independent of age, the severity of COVID-19 in the acute phase, or hospitalization^4,5^. Due to these heterogeneities, the exact pathophysiology of the disease and its neurological sequelae are still unknown, although some mechanisms have been proposed. Therefore, given that it is vague which pathway should be specifically targeted for the treatment of fatigue, a few studies introduced drug treatment options for post-COVID-19 fatigue. Health supplements ImmunoSEB (systemic enzyme complex) and ProbioSEB CSC3 (probiotic complex) with over 90 percent reduction of patients' fatigue showed promising results^24^. However, studies on various medications and populations are still necessary.

Considering the possible shared pathophysiology of long COVID-19 with CFS, treatments for these diseases are one of the solutions for the treatment of post-COVID-19 neurological consequences. However, previous pharmacological and non-pharmacological treatments for CFS have shown inconsistent results. Prescribing medicines used to relieve fatigue from diseases such as cancer and multiple sclerosis are another recommended solution for post-COVID-19 fatigue^25^. Amantadine's effectiveness in relieving MS-related fatigue has been frequently shown^26–28^, and its prescription is recommended by the German MS Society^29^ as well as in clinical practice guidelines published by NICE^30^. Given the high incidence rate of post-COVID-19 fatigue^9,31^ and lack of treatment options^24^, the satisfactory result obtained in our study with treatment with Amantadine is considerable. However, to our knowledge, there is no study investigating the effect of Amantadine on fatigue in post-COVID-19 patients to compare their results with ours.

Abnormalities of the central nervous system (CNS) and muscles might be related to fatigue by several mechanisms, although none of them are definite^32^. Dopamine and GABA are two dominant neurotransmitters that have important roles in central fatigue^33^. Impairment of GABA-ergic inhibition, which points to the central fatigue mechanism has been shown in patients with post-COVID-19 fatigue^34^. The GABA,dopamine, and the N-methyl-D-aspartate (NMDA) regulatory properties of Amantadine are previously documented in the literature^27,35–37^ and likely affected the reduction of COVID-19 fatigue observed in the present trial. In addition to the fatigue-relieving effect of Amantadine shown in our study, it is of benefit in other ways. Amantadine has some anti-inflammatory properties that can be used in the treatment process of acute COVID-19 diseases^38^. Moreover, Amantadine by improving cognitive impairment, which is common in post-COVID-19 patients, with the effect of mental arousal can enhance the suppressed respiratory response to hypoxia and hypercapnia^39,40^. Another worth mentioning is that a two-week Amantadine consumption had tolerable transient side effects, in contrast to an eight-week treatment regimen with Amantadine for chronic fatigue syndrome, which half of the patients could not tolerate and discontinued taking^41^.

Although our study investigated patients with post-COVID-19 fatigue at an acceptable time point with detailed inclusion and exclusion criteria, there are some limitations worth noting. This study was not double-blinded, and we did not use a placebo for the control group. Therefore, the effect of placebo and blinding were not considered in this study and could affect our final results. In addition, we did not limit conventional rehabilitative interventions for patients included in this study, although we had a control group, and this situation was the same for all patients. Thus, we cannot say to what extent the advantages of Amantadine are distinct from or complementary to rehabilitation. Moreover, due to the simple randomization method that we used for allocating patients some features including age and BMI were not statistically similar in both groups. Evidence showed that older age is not correlated with long-COVID fatigue^42^. However, we limited patients' age to young and middle-aged to eliminate any possible effects. Previous research showed BMI > 30 is associated with impaired general health recovery, and a high BMI is known as a risk factor for post‐acute sequelae of COVID‐19. In this study both means of BMI in groups were less than 30, However, the control group had a significantly higher BMI^43^. Therefore, future studies could employ more sophisticated randomization techniques, such as stratified randomization, ensuring better balance in these demographic factors for a more robust and generalized analysis. Although patients showed significant improvement in a two-week course of treatment with Amantadine, we did not follow up with them to investigate its efficacy persistence or recurrence of fatigue. To address these limitations in future research, we recommend incorporating a double-blinded design with a placebo control, imposing stricter control over rehabilitation interventions, implementing more sophisticated randomization methods to achieve balanced baseline characteristics, and conducting long-term follow-up assessments to evaluate the enduring effects of Amantadine on post-COVID-19 fatigue. By addressing these methodological concerns, future studies can enhance the robustness and applicability of findings in this important area of research.

In conclusion, our study demonstrated that consuming Amantadine has a favorable effect on relieving post-COVID-19 fatigue. Our results reveal the safety and tolerability of two-weeks treatment with Amantadine in post-COVID-19 patients. We recommend well-designed double-blind, randomized studies with placebo and larger sample sizes to validate our results.

## Method

This randomized, open-label clinical trial was conducted on randomly selected patients from a pool of patients who came to the internal outpatient clinic of Shahada-E-Tajrish Hospital in the fall of 2022 with complaints of post-COVID-19 fatigue. Patients were included in this study if they had been diagnosed with COVID-19 with confirmed RT-PCR-positive results, had complaints of fatigue 30 to 60 days after the onset of the COVID-19 symptoms, had the willingness and informed consent to participate in the trial, and were between 20 and 50 years of age. It is worth noting that this study was conducted before any of the participants were vaccinated against COVID-19. Also, The patients were treated for COVID-19 according to Iran's national guideline for diagnosing and treating COVID-19^44^.

Exclusion criteria were reinfection of COVID-19, history of psychiatric diseases, suffering from psychotic disorders, anxiety disorders, and major depression during the study, substance abuse in the last four months, taking antidepressants or corticosteroids during the last six weeks, taking psychostimulant drugs, unstable medical condition, cognitive disorders, and confusion, withdrawal from participating in the study, history of the rheumatological disease, getting cancer and malignancy, advanced chronic diseases (heart failure, liver failure, kidney failure, etc.), edema of organs, untreated hypertension, untreated hypogonadism, untreated hypothyroidism, untreated anemia, pregnancy and breastfeeding, convulsions, dyspnea, post-COVID-19 encephalopathy, the intolerability of taking Amantadine due to side effects, and lost to follow-up.

Of the 83 patients included in the study, 17 were initially excluded due to having at least one of the exclusion criteria. Recruited participants were assigned randomly (using simple randomization with a 1:1 ratio) to either the Amantadine receiving (n = 33) or control groups (n = 33). The intervention group received Amantadine 100 mg orally twice daily in the morning and evening for two weeks, but the control group received no treatment. The study was designed open-labeled; Therefore, all the participants and examiners knew the treatment allocation. During the trial, patients were restricted from using any other medication but were not limited to conventional rehabilitation therapies. No changes were made to the approved protocol after the trial began.

All patients completed the Persian version of the Fatigue Severity Scale (FSS) questionnaire at the beginning of the study and two weeks later. The validity and reliability of the questionnaire were confirmed by Shahvaroqi and his colleagues in 2013^23^. In the first part of this questionnaire, there is a Visual Analog Fatigue Scale (VAFS) in which the patients give a score from 0 to 10 to the severity of their fatigue (0 means no fatigue, and 10 means the highest level of fatigue). The second part of that consists of 9 questions, each question is given a minimum of 1 point (complete disagreement of the patient) to a maximum of 7 points (complete agreement of the patient); at the end, the scores of the patient are added up. The basic information, including age, sex, height, weight, and history of hospitalization due to the most recent COVID-19 infection, was recorded for each patient. Common side effects of Amantadine (including headache, dizziness, dry mouth, convulsions, and peripheral edema) were also recorded for Amantadine-receiving patients.

The minimum calculated sample size to achieve a power of 90% with an α = 0.05, and beta = 0.1 was 42 (21 for each group). A total of 66 subjects were recruited, considering potential dropouts due to data missing or protocol violations during the study period. Continuous data were expressed as mean ± standard deviation and categorical data were illustrated as frequencies and percentages. The differences between the two groups in continuous and categorical data were analyzed using the independent t-test and the Chi-square test, respectively. P-values lower than 0.05 were considered statistically significant. Data analysis was performed using SPSS software version 16.

In this study, all methods were carried out in accordance with applicable guidelines, rules, and regulations and was started after the approval of the Shahid Beheshti University of Medical Sciences ethics committee and after obtaining the code of ethics (IR.SBMU.MSP.REC.14001555). and the trial protocol can be accessed at clinicaltrials.gov with a trial registration number of NCT05667077 on 28/12/2022. Also, Written consent was obtained from all the participants in this study.

## Data Availability

The data supporting the results of this manuscript are available from the corresponding author upon reasonable request.
